# Effects of Sanitizers on Microbiological Control of Hatching Eggshells and Poultry Health during Embryogenesis and Early Stages after Hatching in the Last Decade

**DOI:** 10.3390/ani12202826

**Published:** 2022-10-18

**Authors:** Gabriel da Silva Oliveira, Concepta McManus, Cristiane Batista Salgado, Vinícius Machado dos Santos

**Affiliations:** 1 Faculty of Agronomy and Veterinary Medicine, University of Brasília, Brasília 70910-900, Brazil; 2 Laboratory of Geosciences and Human Sciences, Federal Institute of Brasília—Campus Brasília, Brasília 70830-450, Brazil; 3 Laboratory of Poultry Science, Federal Institute of Brasília—Campus Planaltina, Brasília 73380-900, Brazil

**Keywords:** eggshells, embryonic health, hatchery, hatching eggs, microbiological safety, natural materials, poultry farm, poultry production, sanitizers, synthetic chemical materials

## Abstract

**Simple Summary:**

Poultry systems, especially conventional comprehensive production systems to meet the global demand for eggs and meat, are constantly challenged by pathogens, requiring intense sanitary practices. Operations, including the sanitization of hatching eggs, can employ synthetic chemical sanitizers as well as natural plant extracts to minimize the microbial challenge. As the application of formaldehyde sanitizer in hatching eggs cannot be justified in terms of safety for embryonic and human health, studies are underway to assist the industry in adopting new alternative sanitizers. This review aims to evaluate the effects of different sanitizers on the microbiological quality of hatching eggshells and poultry health during embryogenesis and early stages after hatching.

**Abstract:**

The sanitization of hatching eggs is the backbone of the hygienic–sanitary management of eggs on farms and extends to the hatchery. Poultry production gains depend on the benefits of sanitizers. Obtaining the maximum yield from incubation free of toxic sanitizers is a trend in poultry farming, closely following the concerns imposed through scientific research. The toxic characteristics of formaldehyde, the primary sanitizer for hatching eggs, are disappointing, but it is a cheap, practical and widely used antimicrobial. To overcome this shortcoming, multiple synthetic and natural chemical sanitizers have been, and continue to be, tested on hatching eggs. This review aims to evaluate the effects of different sanitizers on the microbiological quality of hatching eggshells and poultry health during embryogenesis and early stages after hatching.

## 1. Introduction

In poultry, embryonic mortality from pathogenic microbial infection is preventable through simple, cheap and efficient preventive guidelines. In most countries, the sanitization of hatching eggs is the primary countermeasure to the attacks of pathogenic microorganisms on the embryo. Studies have shown that sanitizing hatching eggs with synthetic products such as hydrogen peroxide [1] and natural products such as clove essential oil [2] reduced the pathogenic microbiota in eggshells and increased the percentage of hatched chicks. These active materials are non-toxic, non-corrosive and non-damaging to the eggshell. However, unsatisfactory effects such as possible severe toxicity in embryos that led to their death were reported in eggs sanitized with formaldehyde [3]. Microfragments were found in the cuticle and the vertical crystalline layer in eggs sanitized with peracetic acid [4], and reduced hatchability was found in eggs sanitized with propolis [5].

The beneficial and non-beneficial effects of sanitizers in hatching eggs result from synchrony (favorable) or non-synchrony (unfavorable) factors, such as concentration and application time [4,6]. As mentioned earlier, it is clear that sanitizers, when applied to hatching eggs under certain conditions, can generate a repertoire of adverse effects that affect embryonic development. Embryonic health is undoubtedly an important aspect that influences the entire poultry sector. It is through a healthy embryo that a healthy chick will be born. In turn, if handled properly, this chick will become a healthy broiler that will reach the consumer’s table without undue influence on human health. At the same time, the poultry chain experiences significant economic gains for maintenance and growth. However, no sanitizers should be definitively rejected before being fully and continuously evaluated unless the compound is known to be lethally toxic to the point that humans cannot manipulate it with personal protective equipment. Human health must be a priority over all matters considered when choosing a sanitizer for hatching eggs.

Formaldehyde is the primary sanitizer in the routine sanitization of hatching eggs on European poultry farms (for example, Germany and Poland), as well as in Brazil and Egypt, among other countries [2,7,8,9,10]. However, it has genotoxic and cytotoxic properties [11] that subject poultry farmers and chicken embryos to a high risk of hazardous chemical exposure and possible irreversible bodily harm. Indoors, a short exposure not exceeding 0.1 mg/m^3^ (0.08 ppm) of formaldehyde is recommended to avoid damage to human health [12]. Cadirci [13] reported that the concentration required to reduce practically 100% of the microbial load of hatching eggshells is at least 600 mg/m^3^ (489 ppm) of formaldehyde, which is an excessively high concentration when compared to those recommended for human exposure. Therefore, formaldehyde needs to be removed from the routine sanitizing of hatching eggs.

There is a versatile repertoire of synthetic and natural sanitizing formulations for hatching eggs that have contributions from researchers dedicated to studying this line of research in various parts of the world. However, are these formulations able to meet the safety tripod (eggshell microbiological, embryonic and human health) at the oviposition–hatch interface? The compilation of this information is vital for helping the industry by showing it the potential products that can replace formaldehyde once and for all because the trend is for formaldehyde to be banned entirely from poultry farming. This review aims to evaluate the effects of different sanitizers on the microbiological quality of hatching eggshells and poultry health during embryogenesis and early stages after hatching.

## 2. Eggshell and Its Contamination

**Figure 1:**
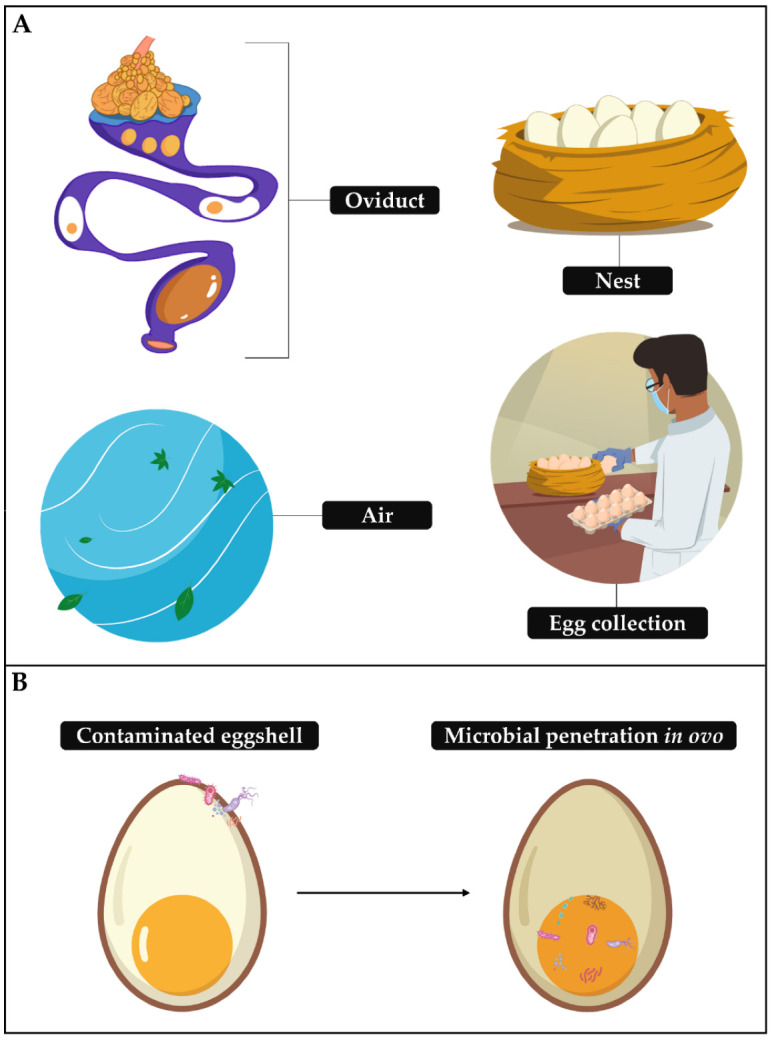
(**A**) Some sources of egg contamination in pre-incubation; (**B**) horizontal transmission of microbes in eggs.

The eggshell is a physical, physiological and immunological protective surface that morphologically and functionally regulates the health of the embryo and supports its development through structural impermeability to pathogens and the expression of proteins that mobilize an antimicrobial response to pathogens [14,15]. Disturbances in antimicrobial functions of the shell by effects on its structure, as well as the resistance and motile capacity of some microorganisms [15,16,17,18] and exposure time of the shell to the microorganism [19,20], are possible causes of horizontal transmission of pathogens (shell–embryo) (Figure 1B), inducing infectious and inflammatory processes. Pathogens such as *Escherichia coli*, *Klebsiella*, *Micrococcus*, *Proteus*, *Pseudomonas*, *Staphylococcus* spp. and *Salmonella* Enteritidis may be associated with egg penetration and embryonic mortality [17,21,22,23]. The sanitation process on the farm is continuously controlled to avoid this burden on embryonic health, chick fatality and eggshell contamination by fungal and bacterial organisms. The latter can be favored by the microclimate on farms and hatcheries [24] and persists from pre-lay to pre-hatch (Figure 1A) [25].

## 3. Sanitizers and the Sanitization of Hatching Eggs

### 3.1. Articles and Search Criteria

Google Scholar was searched using the following keywords: hatching egg sanitization, hatching egg sanitizers and hatching egg disinfectants, in that order. The search process included papers published (between January 2012 and May 2022) in peer-reviewed journals published in English. The first 20 papers were considered for each keyword search, totaling 600 papers. For each year, 10 papers were selected (the first 10 papers found in the search order). A total of 120 papers could have been included. However, after analyzing the title and abstract of the 600 papers, only 69 papers were reviewed, as they studied and evaluated sanitizers for hatching eggs. Review articles on the topic published in this period or other research papers that did not meet all the search criteria were considered to reinforce the discussion.

**Figure 2:**
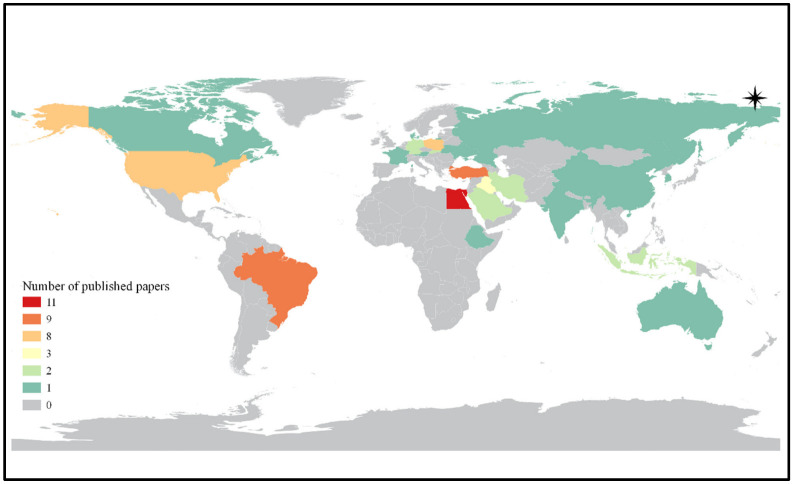
Number of published papers in each country. Countries in the same color have the same number of published papers.

The research studies of papers from the search process were carried out mainly in Egypt, Brazil and Turkey (Figure 2), and the papers were published primarily in *Poultry Science*, *Egyptian Poultry Science Journal* and the *Journal of Applied Poultry Research*.

### 3.2. Objective, Optimal Timing and Methods for Sanitizing Hatching Eggs

**Figure 3:**
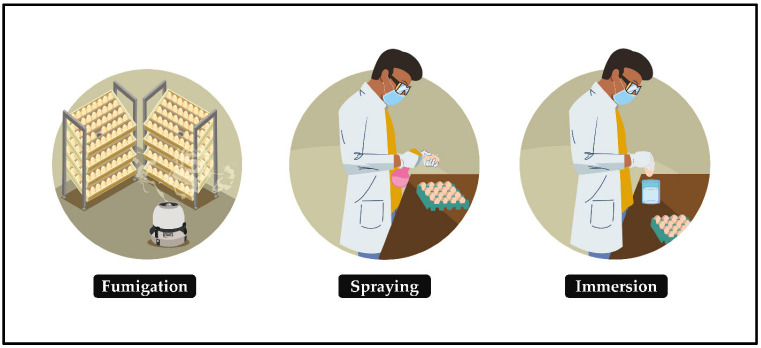
Main methods of sanitizing hatching eggs.

Egg contamination triggers an embryonic health crisis and threatens the world’s poultry economy. This state of affairs can be alleviated by sanitizing hatching eggs, a relatively simple protocol in which the eggs must be submitted, soon after collection, to intervention in the high proliferation of pathogens in the eggshell and their possible mobility to the microenvironment of embryonic development, making the egg suitable for generating a chick. The ideal time to sanitize hatching eggs is up to 30 min after oviposition or collection (if it is immediate) [26,27,28]; otherwise, the probability of having no effect or worsening production results is very high. This is corroborated in [29], which reported improved hatchability of eggs sanitized immediately compared with those sanitized six hours after laying, probably due to microbial penetration. In this protocol, the contact of the sanitizer with the eggs occurs through gaseous or indirect means and by liquid or direct means (Figure 3):

- Fumigation: the release of sanitizing vapors on the surface of hatching eggshells in an enclosed space.
- Spraying: the dispersion of a sanitizing mist on the surface of hatching eggs.
- Immersion: the act of immersing hatching eggs in sanitizer until there is an interaction between them.

The use of each method is based on the size of the production system, number of eggs produced daily, costs and availability of equipment and facilities, type of sanitizer, number of professionals involved in the process and the specific limitations of each method.

### 3.3. Formaldehyde

Formaldehyde (liquid or gaseous; also called paraformaldehyde-polymerized phase) has been linked to reduced eggshell microbiota and increased hatchability percentage. There are also reports that it did not affect any of these variables (Table 1). Nevertheless, it is also associated with reports of toxicity and permanent harmful damage to embryos and chicks when applied to hatching eggs (Table 1). Although these effects depend on the concentration, length of time and method of application of formaldehyde and the period in which the egg is exposed [13], formaldehyde itself is carcinogenic because it impairs and inhibits DNA repair [30]. Therefore, its use is unjustifiable regarding embryonic life safety, health and protection. Poultry production should value lower risks to bird life (whether during development or after hatching), which will benefit the highest priority condition of preserving human health. Given the possible future restrictions on using formaldehyde in the poultry industry, other sanitizers must be readily available and approved by competent bodies to meet the global poultry demand.

## 4. Sanitizers and Their Effects on the Microbiological Quality of Hatching Eggshells and the Health and Survival of Poultry in the Developmental Phase and Early Period after Hatching

The health and survival of embryos and chicks remain the most vital issues for the constant advancement of industrial poultry. This is due to the presence of dangerous microbial agents and the use of risky sanitizers for hatching eggs, representing unquestionable concern for the safety of these animals. In the last 10 years, much research has been conducted on the manufacturing and evaluation of sanitizers to minimize risks during embryogenesis and post-hatch (Table 2). The aim is for these sanitizers to provide vitality and supplements to support the poultry’s quality of life during their development and further growth in the production system. Thus, the effects of sanitizers on eggshell microbiology and hatchability were reviewed (Table 2). Based on the studies reviewed, synthetics stand out over natural sanitizers in the number of published studies. However, when it comes to reducing the microbial load of the shells and increasing hatchability, the positions are reversed, as natural sanitizers present better results than synthetic ones (Table 2). Hatchability and eggshell microbial level are partially capable of predicting health and fully predicting embryo survival and level of risk of damage by pathogens, respectively. They are also associated with production profitability [47]. Studies must be complemented with other analyses, including quality, microbial counts, blood constituents and organ development during embryogenesis and post-hatch, to ensure health and survival.

Among the synthetic chemical sanitizers, hydrogen peroxide, ozone and Virkon S were most commonly tested. Hydrogen peroxide is a reactive oxygen type that exerts antimicrobial activity by inducing oxidative damage to cell DNA [87]. Ozone is a strong oxidant that exhibits antimicrobial characteristics by degrading cellular constituents, impairing their metabolic activity [88]. Virkon S is a combined formulation that includes peroxygen compounds, with antimicrobial action associated with cell wall damage and inhibition of enzymatic systems [89]. These three sanitizers appear to have a safety profile for humans [89,90,91]. Most studies that evaluated hydrogen peroxide reported the ability to reduce eggshell microbial load with almost no damage to hatchability (Table 2). The effectiveness of ozone in reducing eggshell microbial load is still dubious based on the studies reviewed, as half reported no reduction. On the other hand, only 20% reported a significant adverse effect on hatchability, the same ones that reported reduced microbial load. Therefore, the lower hatchability was possibly a response to the toxic action of ozone on the embryo, not seeming to be a good option to sanitize hatching eggs. Regarding the control of the eggshell microbial load, Virkon S performed very well, as demonstrated in all studies, and had no record of significant loss in hatchability. Other examples that may be viable for hatching eggs are ammonium compounds [64], peracetic acid [75], nanosecond electron beam [66], low-energy electron irradiation [38] and ultraviolet radiation [83].

Among the natural sanitizers for hatching eggs, essential oils, volatile liquids produced in flowers, leaves, fruits, seeds, stems and roots [92]; propolis, a resinous product produced by bees using resins and other plant substances [93,94]; and garlic, which is a herb with bulbous flowering [95], are the most tested materials. These have more beneficial than harmful characteristics in terms of antibacterial and antifungal effects and production rates (Table 2), supported by three recently published reviews [25,27,28]. Propolis can improve hatchability by up to 11% [28], and essential oils by up to 12.59% [27]. There are no negative records of garlic in hatchability [25]. The effects of these compounds on eggshell microbial reduction ultimately influence an increase in hatchability. They kill bacterial and fungal pathogens by fully compromising the cell membrane/wall, leading to cell dysfunction and loss [96,97,98,99]. These results, added to the recognized safety of most natural compounds, are essential for preparations of natural origin intended for hatching eggs to acquire a consensual reputation that will be useful for their insertion and permanence in commercial practice to sustain the sanitation management of hatching eggs. They will also be well accepted in free-range, organic and agroecological poultry farming. Other examples include live yeast, vinegar and alcoholic extract of eucalyptus, which showed potential as alternatives as sanitizers for hatching eggs [62,74,77].

The degree of pathogenicity and the concentration of eggshell microorganisms are key considerations in embryo infection, particularly in yolk sac infection [21,25]. If the yolk sac becomes infected, the embryo dies or survives after hatching and remains infected (the microorganism causing the infection (e.g., *Escherichia coli*) can remain for months). Clinical signs include swelling, edema and redness, in addition to limited movement due to abdominal distention, which negatively affects weight distribution, causing balance disturbance. This infectious framework will result in the deprivation of nutrients and maternal antibodies and the absorption of toxins [100]. Therefore, prevention through the application of sanitizers to eggs is the way forward. Upadhyaya et al. [57] reported that essential oil substances, trans cinnamaldehyde and eugenol, reduced *Salmonella enterica* serovar Enteritidis (inoculated on the surface of eggs) to undetectable levels in embryos after being applied to eggshells. The rate of embryonic *Escherichia coli* infection can be minimized in eggs sanitized with Virkon S [3]. Mousa-Balabel et al. [29] reinforced that contaminated hatched chicks are reduced when eggs are efficiently sanitized with propolis. In eggshells experimentally contaminated with *Salmonella* (primary poultry isolate of *Salmonella* Typhimurium), the sanitizer combining hydrogen peroxide and ultraviolet irradiation ensured that this microorganism was undetectable in chicks up to two weeks post-hatch [60]. This is due to the potential of many sanitizers to provide ongoing antimicrobial protection that restricts microbial penetration. Li et al. [68] experimentally inoculated nalidixic acid-resistant *Escherichia coli* (isolated from broiler digestive tract) into hatching eggshells. They indicated that lysozyme prevented this microorganism’s penetration into the egg’s internal environment. This reduces the risk of bacterial infection for embryos and chicks during the early stage of their life, supported by the significant reduction in *Escherichia coli* in the yolk sac. The hatching egg must also be of quality to minimize cracks or shell breaks, reduce incubation residues and infection of birds and increase immunological resistance [78]. In addition, litter eggs should be avoided, as the dirtier the shell, the greater the possibility of containing more pathogenic microorganisms [63].

Sanitizing hatching eggs can optimize embryonic and chick development (based on body weight, organs and length) as well as chick blood hematology and immunity, in addition to microbiological protection of the embryo and chick. These effects have been reported with sanitizers based on garlic oil [67], live yeast [62] and vinegar [74]. Other reports were also described. Sanitizers based on hydrogen peroxide (Hydro-Clean), ammonium compounds (Amino-Steril), peracetic acid (Oxydion) and aldehydes (Viron FF) promote a low frequency of embryonic defects and death, discarding toxic and teratogenic effects [64]. Mousa-Balabel et al. [61] reported that eggs sanitized with Virkon S did not generate weak chicks (inability to hatch) or chicks with incomplete feathers and distorted and wet beaks. Cantu et al. [72] reported the best percentage of hatching ducklings without defects after sanitizing was with hydrogen peroxide plus ultraviolet light. Sanitizers such as Polydez (which contains hydrogen peroxide, benzalkonium chloride, cocamidopropyl betaine, neonol and other components) and Virosan (which contains benzalkonium chloride, glutaraldehyde and excipients) did not harm the development of poultry in the embryonic and post-hatch period [78]. In vitro and in vivo tests performed by Patrzałek et al. [79] confirmed that sanitizing eggs with Dergall (organomodified trisiloxanes) is not toxic to chicken embryos. Oliveira et al. [2] demonstrated that eggs sanitized with clove essential oil improved the physical quality of chicks. This same result was found when the eggs were sanitized with oregano juice [46]. No harmful effects on organ development during embryogenesis and post-hatch were reported in eggs sanitized with clove essential oil [10]. Bekhet and Sayed [82] observed that treating eggs with essential oregano oil did not cause malformations in embryos, benefiting them by restoring their antioxidant balance. Gholami-Ahangaran et al. [3], Batkowska et al. [43] and Oliveira et al. [10] reported improvement in the survival percentage of chicks from eggs sanitized with Virkon S, propolis and clove essential oil, respectively, in the first days of post-hatch life.

Sanitizers capable of inducing damage that prematurely interrupts the development and growth of poultry or that reduces their quality of life have been reported. In the study of Shafey et al. [18], low hatchability was associated with the sanitization of eggs with ultrasonic waves. According to this report, embryos exposed to these waves can develop abnormally. Low hatchability has also been described in hatching eggs sanitized with lemongrass and pedestrian tea essential oils [6]. Mousa-Balabel et al. [61] noted that eggs sanitized with hydrogen peroxide recorded weak chicks and a high percentage of omphalitis, and eggs sanitized with TH4 recorded weak chicks with distorted beaks. Oliveira et al. [5] observed that the few chicks that managed to hatch from eggs sanitized with propolis were super-hydrated. Wlazlo et al. [44] showed that ozone has a toxic profile for interrupting embryonic development, justified by the high mortality rate recorded. These studies say much about the sensitivity of embryos to the stressful effects of sanitizers in hatching eggs. Hasyim et al. [101] found a numerical increase and decrease in hatchability when eggs were sanitized with cherry leaf extract at low and high concentrations, respectively, justifying the reduction in hatchability due to the occlusion of the shell pores. Chung et al. [69] reported that the use of chlorine dioxide at low concentrations has no adverse effect on hatchability as seen at high concentrations. The side effects of chlorine dioxide on the embryo were associated with low temperature, high concentration of sanitizer and contact time with the egg [102]. Reducing incidents of sanitizer toxicity can be achieved by adequately balancing the intrinsic factors linked to efficiency that influence toxicity, such as efficiency, safety, minimum concentration and shorter contact time.

Progress in sanitizer evaluation offers some possibilities and future avenues of application at the commercial level. Hydrogen peroxide and Virkon S are among the synthetic chemicals, and essential oils, propolis and garlic are among the natural products due to their antimicrobial efficiency and little or no adverse effect recorded on embryos and chicks, in addition to meeting safety requirements for humans. However, we believe that it is necessary to continuously deepen the evaluations carried out (mainly in vivo toxicity analyses at different concentrations) during embryogenesis and post-hatch after sanitizing the eggs with these antimicrobials to find the most suitable, affordable, efficient and safe protocol possible. We need to reinforce the benefits of existing protocols or discard those that, in part, may persistently cause some disadvantages to the process. While hydrogen peroxide, Virkon S, essential oils, propolis, and garlic may meet safety criteria, proper protective clothing and other safety precautions are necessary during exposure.

Despite being a challenge, a problem observed among the studies reviewed is the non-standardization of the time for sanitizing eggs after collection. Some studies performed this outside the ideal timeframe, for example, very close to or during the incubation process. This can negatively affect the process. Oliveira et al. [25] recommended that eggs should be sanitized in the shortest possible time after collection, which also requires speed, to achieve the objective of minimizing in ovo penetration and ensuring the chances of increasing the hatchability rate healthily. Laboratory studies should be complemented with egg sanitization repetitions on commercial farms. If carried out efficiently and adequately after collection, a single treatment should be sufficient until hatching, keeping all other surfaces where the eggs pass clean and sanitized.

## 5. Conclusions

Knowing that the abusive and poisonous use of formaldehyde fumigation for hatching eggs cannot be underestimated, this review demonstrates that research advances in the last decade have defended, at different levels, powerful safe alternatives based on synthetic and natural products. In addition to their antimicrobial capacity, these substances can mitigate the toxic effects that decrease bird health and survival by respecting the protocols recommended by researchers. This is a big step for the poultry industry, helping to understand and limit the use and availability of formaldehyde towards its total exclusion, making future handling of hatching eggs increasingly free of toxicity.

## Figures and Tables

**Table 1:** Some reports of the effects of formaldehyde on hatching eggs.

**Study (Reference)**	Effect on Eggshell Microbial Count *	Effect on Hatchability *
[31]	Non-evaluated	No effects
[32]	No effects	No effects
[33]	-	No effects
[34]	Reduced	Increased
[35]	Reduced	Non-evaluated
[36]	-	Increased
[37]	Reduced	Non-evaluated
[38]	Reduced	No effects
[2]	Reduced	Increased
[39]	Non-evaluated	Increased
**Study (Reference)**	**Some Reports of Adverse Effects on Embryos and Chicks**	
[40]	Underweight, underdeveloped and malformed embryos.	
[41]	Increased embryonic mortality in the early stage.	
[42,43,44]	Reduced chick survival rate in the first post-hatch week.	
[45]	Increased embryonic mortality in early, mid and late stages.	
[46]	Reduced chick quality score as a result of slow activities and high number of unclosed navels.	

* Effect compared to a negative control (non-sanitized eggs) and in the absence of negative control compared to the other sanitizers tested.

**Table 2:** Reports of the effects of sanitizers on eggshell microbial counts and hatchability.

Study (Reference)	Sanitizer	Effect on Eggshell Microbial Count *	Effect on Hatchability *
[31]	Ozone	-	No effects
[48,49]	Propolis	Reduced	No effects
[50]	PotoClean	Reduced	-
[32]	Orthophenylphenol	Reduced	No effects
	Stabilized hydrogen peroxide + peracetic acid + acetic acid		
	Sodium hypochlorite + chlorine dioxide + sodium chlorite + ozone + water		
[51]	Ethanol	Reduced	No effects
[52]	Propolis	Reduced	No effects
[18]	Ultrasonic waves	-	Increased
[53]	Bac-D	Reduced	No effects
[54]	Quaternary ammoniums + bronopol + biguanide	Reduced	-
	Quaternary ammoniums + polyhexamethylenebiguanide hydrochloride moiety		
	Hydrogen peroxide		
	Ammonium chlorides + hydrogen peroxide		
	Quaternary ammoniums		
[34]	Propolis	Reduced	Increased
	Thyme essential oil		
[55]	Quaternary ammoniums + a polyhexamethylenebiguanide hydrochloride moiety	Reduced	Reduced (commercial facility testing) and no effects (lab testing)
[56]	Biosentry 904	-	No effects
	Egg-Washer-Pro		
	Virkon S		
[57]	Trans-cinnamaldehyde	Reduced	-
	Eugenol		
[7]	Ultraviolet light	-	No effects
[58]	Ultrasonic waves	Reduced	No effects
[40]	Hydrogen peroxide	Reduced	-
	Sodium chloride		
	Betadine		
	Virkon S		
	Cumin essential oil		
	Oregano essential oil		
	Cumin + oregano essential oils		
[59]	Hydrogen peroxide + ultraviolet irradiation	Reduced	-
[35]	Sodium dichlorocyanurate	Reduced	-
	Hydrogen peroxide	Reduced	
	Electrolyzed oxidizing water	No effects	
[3]	Virkon S	-	No effects
[29]	Propolis	Reduced	-
	TH4		
	Virkon S		
[60]	Hydrogen peroxide + ultraviolet irradiation	Reduced	-
[61]	Hydrogen peroxide	Reduced	Increased
	TH4		
	Virkon S		
[42]	Colloidal silver	Reduced	No effects
[62]	Live yeast	Reduced	Increased
[63]	Virocid	Reduced	-
[64]	Amino-Steril	-	No effects
	Oxydion		
	Viron FF		
	Hydro-Clean		
[65]	Volatile pyrazines	Reduced	-
[1]	Hydrogen peroxide	Reduced	Increased
[66]	Nanosecond electron beam	Reduced	No effects
[67]	Garlic oil	Reduced	Increased
[8]	Hydrogen peroxide	Reduced	Reduced
	TH4		
[68]	Lysozyme	Reduced	No effects
[69]	Chlorine dioxide gas	Reduced	No effects
[41]	Garlic extract	-	No effects
[70]	Grapefruit juice	Reduced	No effects
[37]	Ozone	No effects	-
	Ultraviolet light	Reduced	
	Hydrogen peroxide	No effects	
	Peracetic acid	No effects	
[43]	Propolis	No effects	No effects
[71]	Olive oil	-	Reduced
	Albumin		
[72]	Hydrogen peroxide + ultraviolet light	Reduced	Increased
[73]	Noni leaf extract	-	No effects
[6]	Lemongrass essential oil	Reduced	Reduced
	Pedestrian tea essential oil		
	Lemongrass + pedestrian tea essential oils		
[74]	Vinegar	Reduced	Increased
[75]	Ozone	No effects	No effects
	Ultraviolet light	Reduced	
	Hydrogen peroxide	No effects	
	Peracetic acid	Reduced	
[2]	Clove essential oil	Reduced	Increased
[5]	Propolis	-	Reduced
	Clove essential oil		No effects
[76]	Fenugreek seed extract	-	No effects
	Oat seed extract		
	Basil seed extract		
[77]	Eucalyptus alcoholic extract	Reduced	Increased
[78]	Polydez	Reduced	-
	Sterylii AB	No effects	
	Virosan	Reduced	
[79]	Dergall	Reduced	No effects
[9]	Hydrogen peroxide	-	No effects
	Low-energy electron irradiation		
	Peracetic acid		
	Essential oil (not specified)		
[38]	Low-energy electron irradiation	Reduced	No effects
[44]	Hydrogen peroxide	Reduced	No effects
	Ozone		Reduced
[80]	Hydrogen peroxide	-	No effects
[81]	Pulsed ultraviolet light	Reduced	No effects
[82]	Oregano essential oil	-	Increased
[83]	Ultraviolet light	Reduced	-
[39]	Ozone	-	Increased
	Aldekol		
	Virkon S		
[84]	Ozone	Reduced	No effects
[85]	Garlic oil	-	Increased
[46]	Oregano juice	Reduced	No effects
[86]	Slightly acidic electrolysis	Reduced	No effects

* Effect compared to non-sanitized eggs (or water or alcohol control) and formaldehyde (or other positive control). The negative control (non-sanitized eggs) had priority in the comparison. An increase was also considered when the sanitizer was tested at different concentrations or methods and at least one of those concentrations or methods showed improvement. (-) When the tested sanitizer did not have the variable evaluated or when it did, the study did not apply or clarify a statistical analysis and was not compared to a positive or negative control group. Studies that evaluated only formaldehyde were not included in this table, as the focus was on alternatives.

## Data Availability

Not applicable.
